# Osteoinductive Effects of Free and Immobilized Bone Forming Peptide-1 on Human Adipose-Derived Stem Cells

**DOI:** 10.1371/journal.pone.0150294

**Published:** 2016-03-01

**Authors:** Wenyue Li, Yunfei Zheng, Xianghui Zhao, Yanjun Ge, Tong Chen, Yunsong Liu, Yongsheng Zhou

**Affiliations:** 1 Department of Prosthodontics, Peking University School and Hospital of Stomatology, Beijing, China; 2 Department of Oral and Maxillofacial Surgery, Peking University School and Hospital of Stomatology, Beijing, China; 3 National Engineering Lab for Digital and Material Technology of Stomatology, Peking University School and Hospital of Stomatology, Beijing, China; University of California, San Diego, UNITED STATES

## Abstract

Most synthetic polymeric materials currently used for bone tissue engineering lack specific signals through which cells can identify and interact with the surface, resulting in incompatibility and compromised osteogenic activity. Soluble inductive factors also have issues including a short half-live *in vivo*. Bone forming peptide-1 is a truncated peptide from the immature form of bone morphogenetic protein-7 (BMP-7) that displays higher osteogenic activity than full-length, mature BMP-7. In this study, we used a mussel-inspired immobilization strategy mediated by polymerization of dopamine to introduce recently discovered stimulators of bone forming peptide-1 (BFP-1) onto the surface of poly-lactic-co-glycolic acid (PLGA) substrate to form a biomaterial that overcomes these challenges. Human adipose-derived stem cells (hASCs), being abundant and easy accessible, were used to test the osteogenic activity of BFP-1 and the novel biomaterial. Under osteoinductive conditions, cells treated with both BFP-1 alone and BFP-1-coated biomaterials displayed elevated expression of the osteogenic markers alkaline phosphatase (ALP), osteocalcin (*OC*), and *RUNX2*. Furthermore, hASCs associated with poly-dopamine-assisted BFP-1-immobilized PLGA (pDA-BFP-1-PLGA) scaffolds promoted *in vivo* bone formation in nude mice. Our novel materials may hold great promise for future bone tissue engineering applications.

## Introduction

Inductive factors that stimulate the osteogenic differentiation of stem cells play a crucial role in bone tissue engineering, and the search for more effective factors to help bone formation remains on. Bone morphogenetic proteins (BMPs) are members of the transforming growth factor beta (TGF-β) super-family that induce differentiation of stem cells into osteoprogenitor cells to form new bone, and are considered the most potent osteoinductive growth factors available [1, 2]. However, their use is complicated by their difficult and expensive synthesis, and their side effects that include swelling and increased risk of cancer [3, 4]. The observation that truncated peptides exhibit osteoinductive activity offers a potential strategy for solving the problem. Bone forming peptide-1 (BFP-1) is a 15 amino acid peptide (GQGFSYPYKAVFSTQ) derived from the immature form of morphogenetic protein-7 (BMP-7) that displays higher osteoinductive activity towards mouse bone marrow stromal cells (mBMSCs) than does the full-length, mature protein [5]. In mBMSCs treated with BFP-1, alkaline phosphatase (ALP) activity and calcium depositions were enhanced, and expression of CD44, CD47 and CD51 was up regulated. Furthermore, implantation of BFP-1-treated mBMSCs into mice strongly stimulated bone formation during an eight week trial, and BFP-1 also enhanced osteogenic differentiation of human bone marrow-derived mesenchymal stem cells (hBMMSCs) [6].

Human adipose-derived stem cells (hASCs) are widely regarded as promising seed cells for bone tissue engineering [7]. Unlike hBMMSCs, hASCs are more easily harvested from donors in larger quantities and at lower cost. However, contention remains regarding their ability to differentiate into osteoblastic cells and to respond to stimulators, with some researchers claiming they are comparable [8, 9], while others report significant quantitative differences, with factors that greatly influence hBMMSCs having little or no impact on hASCs [10–12]. Given the benefits of hASCs discussed above, we decided to investigate the effects of BFP-1 on hASCs in the present study, using hBMMSCs as controls.

Poly-lactic-co-glycolic acid (PLGA) is a copolymer of poly-lactic acid (PLA) and poly-glycolic acid (PGA) that has been used widely in medicine due to appropriate biodegradation properties and mechanical strength [13, 14], and clinical application has been approved by the FDA [15]. PLGA has high value for use in tissue engineering applications, but also shortcomings including poor hydrophilicity and a low cell adhesion rate [16]. These shortcomings affect the adhesion and proliferation of cells on the surface of the material and disrupt bone formation. In this study, we employed a mussel-inspired immobilization strategy based on dopamine (DA) polymer to immobilize BFP-1 on a PLGA surface. DA is known to form a poly-dopamine film (pDA) on a variety of matrices under alkaline pH conditions via a simple self-polymerization reaction [17], and pDA displays strong adhesion properties and almost no side effects on cellular activity [18]. In this study, we tested the effects of BFP-1 on cell differentiation of hASCs *in vitro*, using hBMMSCs as controls, and the osteoinductive activity of pDA-mediated BFP-1-immobilized PLGA substrates were tested *in vitro* and *in vivo*.

## Materials and methods

### Cell culture and osteogenic induction

Cells (hBMMSCs and hASCs) were purchased from ScienCell (San Diego, CA, USA). All materials were purchased from Sigma-Aldrich (St. Louis, MO, USA) unless otherwise stated. Dulbecco's modified Eagle's medium (DMEM), fetal bovine serum (FBS), 100× penicillin and streptomycin were purchased from Gibco (Grand Island, NY, USA). Cells from three donors at passages 4–6 were used for *in vitro* experiments. For the study on hASCs, all experiments were repeated at least three times using cells from two donors, and the mean value was calculated from three independent experiments. For the study on hBMMSCs, all experiments were also repeated at least three times using cells from another donor, and the mean value was again calculated from three independent experiments. Proliferation medium (PM) comprised fresh DMEM containing 10% (v/v) FBS, 100 U/mL penicillin G and 100 mg/mL streptomycin. PM supplemented with 10 nM dexamethasone, 10 mM β-glycerophosphate and 50 mg/ml L-ascorbic acid constituted osteogenic medium (OM). Culture medium was changed every 3 days.

### Cell proliferation assay

The effects of BFP-1 at different concentrations on the proliferation of hASCs and hBMMSCs were evaluated by cell counting assay using a Cell Counting Kit-8 (CCK8) (Dojindo Laboratories, Kumamoto, Japan). Cells were seeded at 1×10^4^ cells per well in a 24-well plate and cultured in PM or PM supplemented with 1, 2 or 5 μg/ml BFP-1. The relative cell number was determined using CCK8 after 1, 3 and 5 days of culturing, according to the manufacturer's instructions. The OD (absorbance) value of each well was converted to relative cell number using a standard curve.

### Alkaline phosphatase (ALP) activity of BFP-1-induced cells

Cells were seeded in 12-well plates at a density of 5×10^4^ cells per well, divided into four groups after 24 h, cultured in PM, PM containing 1 μg/ml BFP-1 (PM-BFP-1), OM, or OM containing 1 μg/ml BFP-1 (OM-BFP-1). After 7 days of culturing, osteogenic differentiation of cells was measured by ALP activity assays using an ALP kit according to the manufacturer’s protocol. ALP levels were normalized against total protein content as previously described [19].

### Mineralization assays for BFP-1-induced cells

Cells were seeded in 12-well plates and divided into four groups as above. Matrix mineralization was evaluated by staining with AR-S on day 14 after culturing in OM. For quantification of matrix calcification, plates were washed with PBS three times and fixed with ethanol for 30 min, then stained with 1% AR-S in dH_2_O (pH 4.2) for 20 min at room temperature. After staining, wells were washed three times with dH_2_O. To quantify matrix mineralization, alizarin red S-stained cultures were incubated in 100 mM cetylpyridinium chloride for 1 h to solubilize calcium-bound alizarin red S, the absorbance of which was measured at 562 nm [20].

### RT-PCR assays for BFP-1-induced cells

Cells were seeded in 6-well plates and divided into four groups as above. All layers were harvested on 7 and 14 days after osteogenic induction. Total RNA was isolated using Trizol reagent (Invitrogen, Carlsbad, CA, USA) and used for first strand cDNA synthesis using the Reverse Transcription System (Roche, Basel, Switzerland). Real-time quantitative PCR assays were performed according to manufacturer's instructions (Bio-Rad, Hercules CA, USA). Expression of β-actin was used as an internal control. Primers used were as follows:

*RUNX2*, 5'-ATGGGATGGGTGTCTCCACA-3' (forward), 5'-CCACGAAGGGGAACTTGTC-3' (reverse).

*OC*, 5'-CACTCCTCGCCCTATTGGC-3' (forward), 5'-CCCTCCTGCTTGGACACAAAG-3' (reverse).

β-actin, 5'-CATGTACGTTGCTATCCAGGC-3' (forward), 5'-CTCCTTAATGTCACGCACGAT-3' (reverse).

### Surface modification of films and scaffolds

Poly-lactic-co-glycolic acid (PLGA) with a 50:50 copolymer ratio was purchased from Shandong Academy of Pharmaceutical Sciences (Shandong, China). The average molecular weight of PLGA films and 3D scaffolds was 100,000 and 7,000, respectively, and the thickness was 100 μm and 2 mm, respectively. The pore size of scaffolds was 200–300 μm. PLGA films and scaffolds were made for *in vitro* culture and *in vivo* transplantation, respectively. Both were immersed in dopamine solution (2 mg/mL in 10 mM Tris-HCl, pH 8.5, Sigma, St. Louis, MO) and at room temperature for 18 h with shaking, then washed with distilled water in an ultrasonic cleaner to remove unattached DA molecules. For immobilization of BFP-1 (sequence = GQGFSYPYKAVFSTQ; GL Biochem, Shanghai), pDA-coated PLGA substrates were immersed in peptide solution (1 mg/mL in PBS) for 12 h at room temperature, then washed with distilled water to remove unattached peptides [21]. To calculate the efficiency of pDA-BFP-1 binding to the PLGA substrates, 75 μl of supernatant was retrieved from the sample immediately after immobilization and reacted with 25 μl of fluorescamine solution (3 mg/ml in acetone, Sigma) for 15 min at room temperature. The fluorescence intensity of the sample (excitation = 390 nm, emission = 465 nm) was measured using a Multilabel Reader (2300, PerkinElmer, Singapore). The actual concentration of the unattached BFP-1 in each sample was calculated from a standard curve of BFP-1 standards of known concentrations (between 1 μg and 1 mg/ml). After BFP-1 immobilization, engineered PLGA substrates were incubated in physiologically relevant conditions (PBS, 37°C) and supernatants were sampled each day for six days to measure BFP-1 release.

### Surface characterization

Static water contact angles of bare PLGA film, pDA-coated PLGA film, and pDA-BFP-1-immobilized PLGA film were measured by a Dataphysics OCA20 contact angle system (Filderstadt, Germany) at room temperature. X-ray photoelectron spectroscopy (XPS) was performed using an Axis Ultra (Kratos Analytical, U.K.) to compare the chemical composition of films before and after immobilization. Immobilization of peptides was measured using fluorescein isothiocyanate-labeled peptide (FITC-BFP-1), with fluorescence detected by an Eclipse TE300 fluorescent microscope (Nikon, Japan).

The surface morphology of PLGA scaffolds was characterized by field emission scanning electron microscopy using a Hitachi S4800 instrument (Hitachi, Tokyo, Japan). Before observation, samples were washed with phosphate buffered saline (PBS) and fixed overnight in cacodylate buffered 4% glutaraldehyde at 4°C, dried in a Micro Modul YO-230 critical point dryer (Thermo Scientific, Waltham, MA, USA), mounted onto aluminum stubs, sputter-coated with gold, and viewed using a field emission SEM [20].

### Proliferation and adhesion assays of immobilized cells

Cells were seeded at 1×10^4^ cells per well onto pDA-PLGA or pDA-BFP-1-PLGA sterilized films placed in a 24-well plate. Cell proliferation was measured by CCK8 assay after 0, 1, 2, 3, 4, 5, and 6 days of cell culturing as described above.

To observe the morphology and number of adhered cells using a confocal microscope, films were washed with PBS after 12 h of culturing, fixed in 4% paraformaldehyde for 10 min at room temperature, washed twice with PBS and post-fixed in 0.1% Triton X-100 for 7 min at room temperature. Films were then washed three times with PBS and incubated with fluorescein isothiocyanate (FITC)-labeled phalloidin for 25 min at 37°C. Films were washed in PBS three more times and incubated in 5 mg/ml 6-diamidino-2-phenylindole (DAPI) solution for 5 min at 37°C. Films were then transferred to a glass slide and viewed under a Confocal Zeiss Axiovert 650 microscope (Carl Zeiss Microimaging, Oberkochen, Germany) using laser wavelengths of 488 nm (green, FITC-labeled phalloidin) and 405 nm (blue, DAPI-labeled)[20]. Scaffolds used in animal experiments were observed using the same process 12 h after cell seeding.

### Differentiation assays of cells on the engineered materials

Cells were seeded on films as described above, and after 7 days of osteoinduction, ALP activity was determined as previously described. After 14 days of osteoinduction, alizarin red S (AR-S) staining and mineralization assays were performed as above.

### Animal experiments

This study was approved by the Institutional Animal Care and Use Committee of the Peking University Health Science Center (LA2014233), and all animal experiments were performed in accordance with the Institutional Animal Guidelines. The nude mice were purchased from Vital River Corporation (Beijing, China) and allowed free access to water and a maintenance diet, with room temperature at 21±2°C and in a 12-hour light/dark cycle. Implants were divided into six groups: (1) PLGA scaffold only (PLGA), (2) PLGA scaffold coated with pDA (pDA-PLGA), (3) PLGA scaffold coated with pDA and BFP-1 (pDA-BFP-1-PLGA), (4) PLGA loaded with hASCs (PLGA+cell), (5) pDA-PLGA loaded with hASCs (pDA-PLGA+cell), (6) pDA- BFP-1-PLGA loaded with hASCs (pDA-BFP-1-PLGA+cell). PLGA scaffolds were sterilized with 75% ethanol for 1 h before cell seeding [22, 23], washed twice with PBS, and immersed in PM overnight. Next, hASCs were seeded onto PLGA, pDA-PLGA, and pDA-BFP-1-PLGA films at a density of 1×10^6^ cells/ml (4 scaffolds per 1 mL of cell suspension) in a low attachment well and gently rotated for 3 h at 37°C (n = 6 for each group). Cell-loaded scaffolds were cultured in PM for 2 days, and then transferred to OM for 4 days before transplantation. All implants were placed into subcutaneous areas of the backs of 6-week-old male BalB/c nude mice anaesthetized with pentobarbital. At 4 and 8 weeks after implantation, mice were sacrificed by CO_2_ inhalation. Implants were harvested together with their surrounding tissues and fixed using formalin.

### HE staining and immunohistochemistry

All harvested implants were decalcified for 14 days in 10% EDTA (pH 7.4), and specimens were dehydrated and subsequently embedded in paraffin. Sections (5 μm thickness) were stained with hematoxylin and eosin (HE). Osteogenesis was evaluated by immunohistochemical (IHC) staining for RUNX2 and osteocalcin. Primary antibodies (ab93876-Osteocalcin, ab23981-RUNX2, Abcam, Cambridge, UK) were diluted 1:100 and applied overnight at 4°C. The volume of new bone formation was measured using ImageJ and quantified as the percentage of bone volume in the total tissue volume [(bone volume / tissue volume) × 100%]. Bone formation area was confirmed by both HE and immunohistochemical staining.

### Statistical analysis

Results are presented as means ± standard deviations. Data were analyzed by one-way analysis of variance followed by Duncan’s post-hoc test using SPSS 19.0 software (SPSS Inc., Chicago, IL, USA). For all tests, p <0.05 was considered statistically significant.

## Results and Discussion

### Effect of soluble BFP-1 on proliferation and osteogenic differentiation of hASCs in vitro

**Fig 1:**
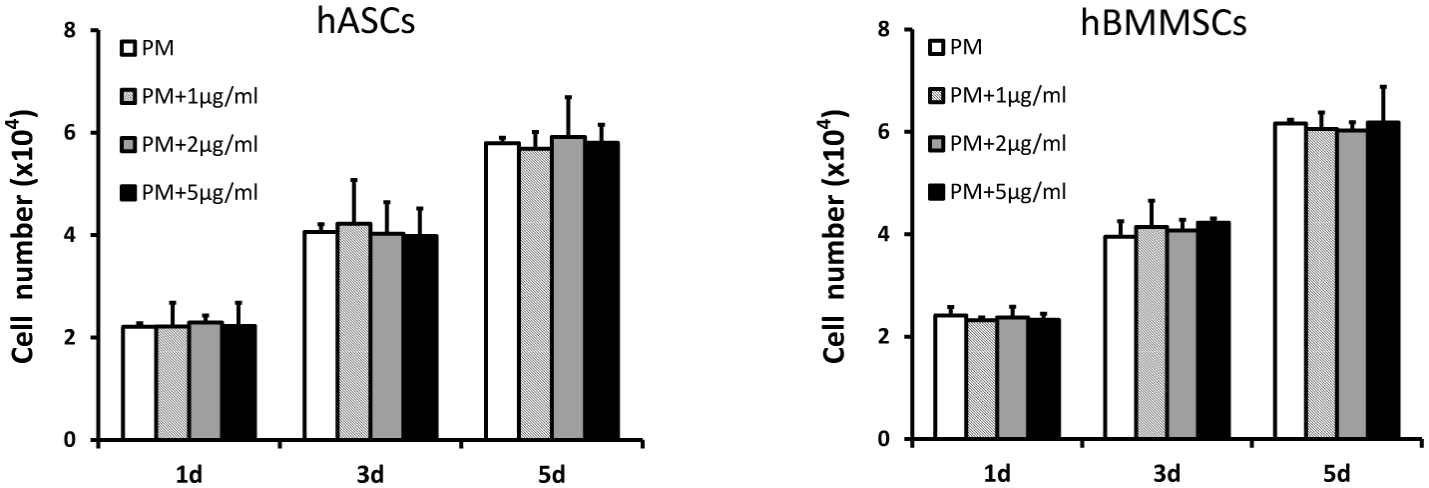
CCK8 assay of hASCs and hBMMSCs cultured with different concentrations of BFP-1. CCK8: Cell Counting Kit-8; hASCs: human adipose-derived stem cells; hBMMSCs: human bone marrow-derived mesenchymal stem cells; BFP-1: bone forming peptide-1. (P >0.05).

To investigate the effect of free BFP-1 on hASCs *in vitro*, assays were performed that involved measuring known osteogenic indices (early osteogenic transcription factor *RUNX2*, middle-stage osteogenesis-related enzyme ALP, middle-and-late-stage osteogenic marker OC, and late-stage osteogenesis assay AR-S) [24] at different time points, with hBMMSCs as controls. CCK-8 assays (Fig 1) revealed no difference between BFP-1 and control groups after 1, 3, and 5 days of culturing at BFP-1 concentrations of 1, 2 or 5 μg/ml, indicating no significant effect on proliferation of hASCs or hBMMSCs (P >0.05). This was in accordance with the previous report [5].

**Fig 2:**
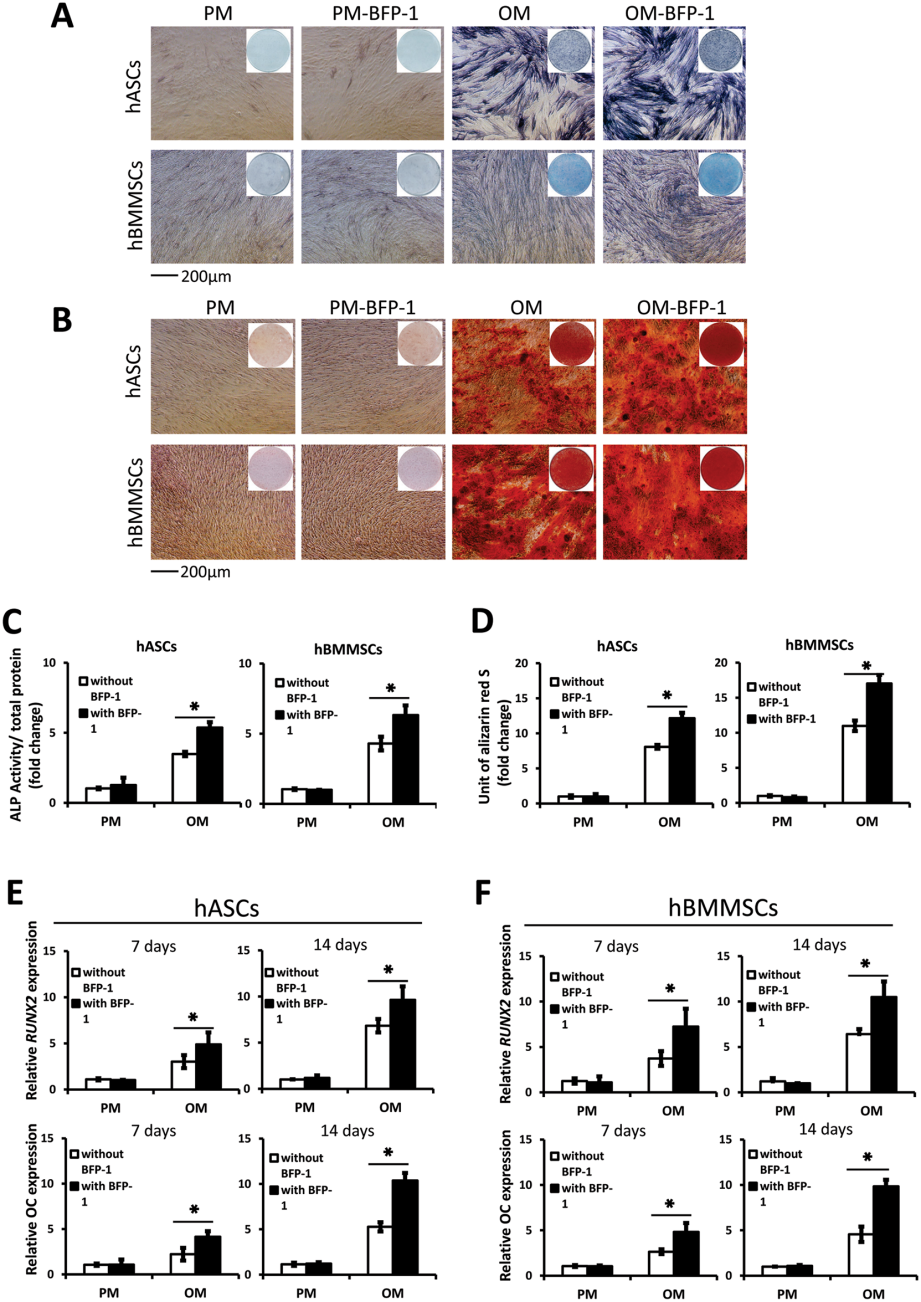
Osteogenic differentiation of hASCs and hBMMSCs treated with free BFP-1 *in vitro*. hASCs: human adipose-derived stem cells; hBMMSCs: human bone marrow-derived mesenchymal stem cells; BFP-1: bone forming peptide-1; PM: proliferation medium; OM: osteogenic medium. Alkaline phosphatase (ALP) activity of hASCs and hBMMSCs (A, C) cultured with or without BFP-1 for 7 days (*: P <0.05). Alizarin Red staining (B) and mineralization assay (D) of hASCs and hBMMSCs cultured with or without BFP-1 at 14 days after osteoinduction (*: P <0.05). Expression of osteogenic genes *RUNX2* and *OC* in hASCs (E) and hBMMSCs (F) cultured for 7 and 14 days after osteoinduction (*: P <0.05).

After 7 days of osteoinduction (OI), the BFP-1 group showed higher ALP activity than control groups (p <0.05; Fig 2A and 2C). After 14 days of OI, AR-S staining and mineralization assays demonstrated that BFP-1 groups underwent more mineralization than control groups lacking BFP-1 (Fig 2B and 2D). After 7 days and 14 days of OI, the relative expression in BFP-1 groups in was higher than control groups in both hASCs and hBMMSCs (P <0.05; Fig 2E and 2F). These results suggest under osteoinductive conditions, BFP-1 promoted osteogenic differentiation to a similar degree in both hASCs and hBMMSCs *in vitro*.

### Surface characteristics of engineered PLGA substrates

**Fig 3:**
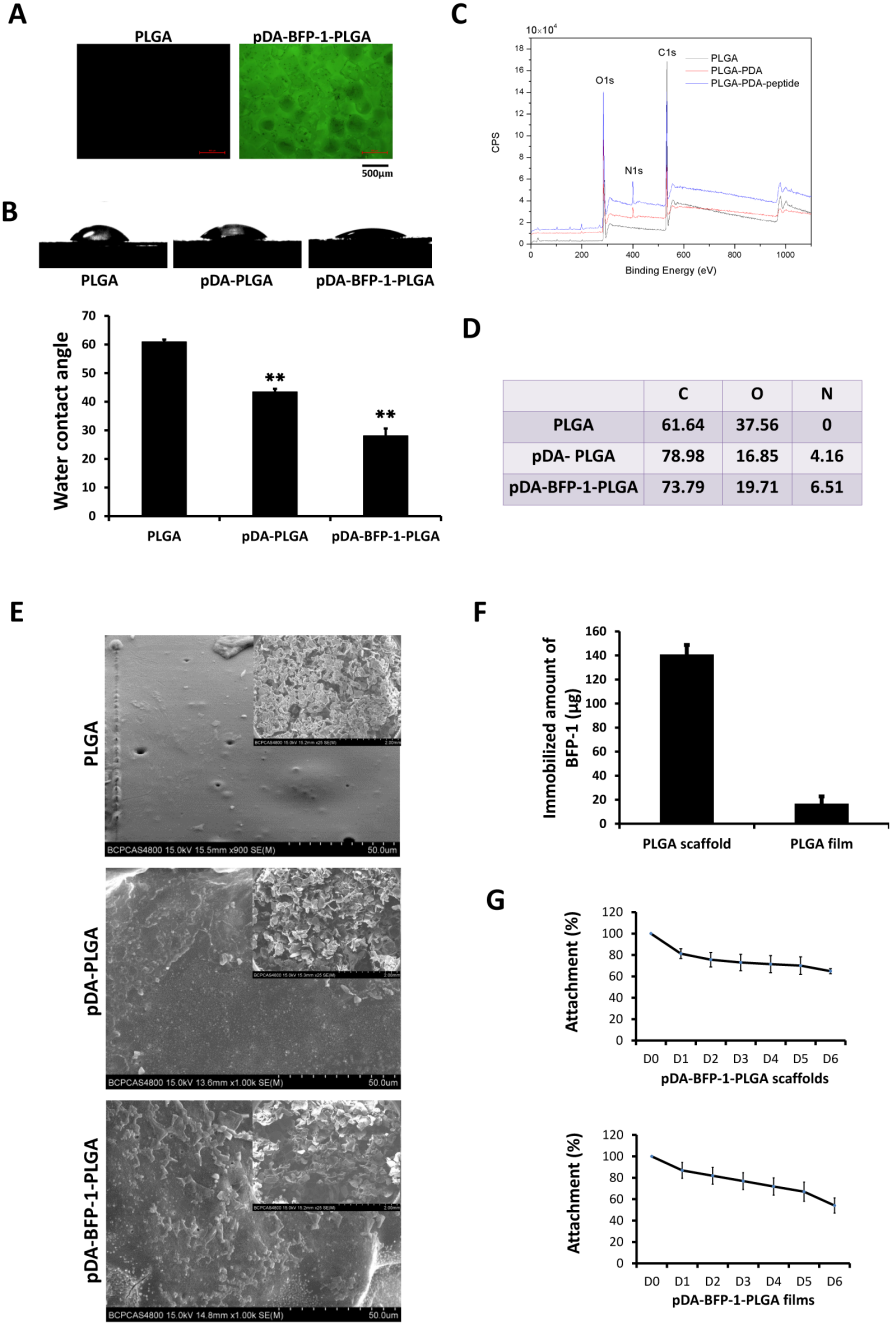
Surface characterization of engineered PLGA substrates. (A) Fluorescent images of PLGA substrates with (right) or without (left) immobilized, fluorescently labeled peptides (FITC-BFP-1). (B) Contact angle measurement of PLGA, pDA-PLGA, and pDA-BFP-1-PLGA films (**: p <0.01). (C) X-ray photoelectron spectroscopy (XPS) analysis of PLGA, pDA-PLGA and BFP-1-pDA-PLGA films. (D) Chemical composition of film surfaces. (E) Scanning electron microscopy of PLGA, pDA-PLGA and pDA-BFP-1-PLGA scaffolds. (F) Amount of immobilized BFP-1 (μg). (G) Release curves of pDA-BFP-1-PLGA scaffolds and films.

**Fig 4:**
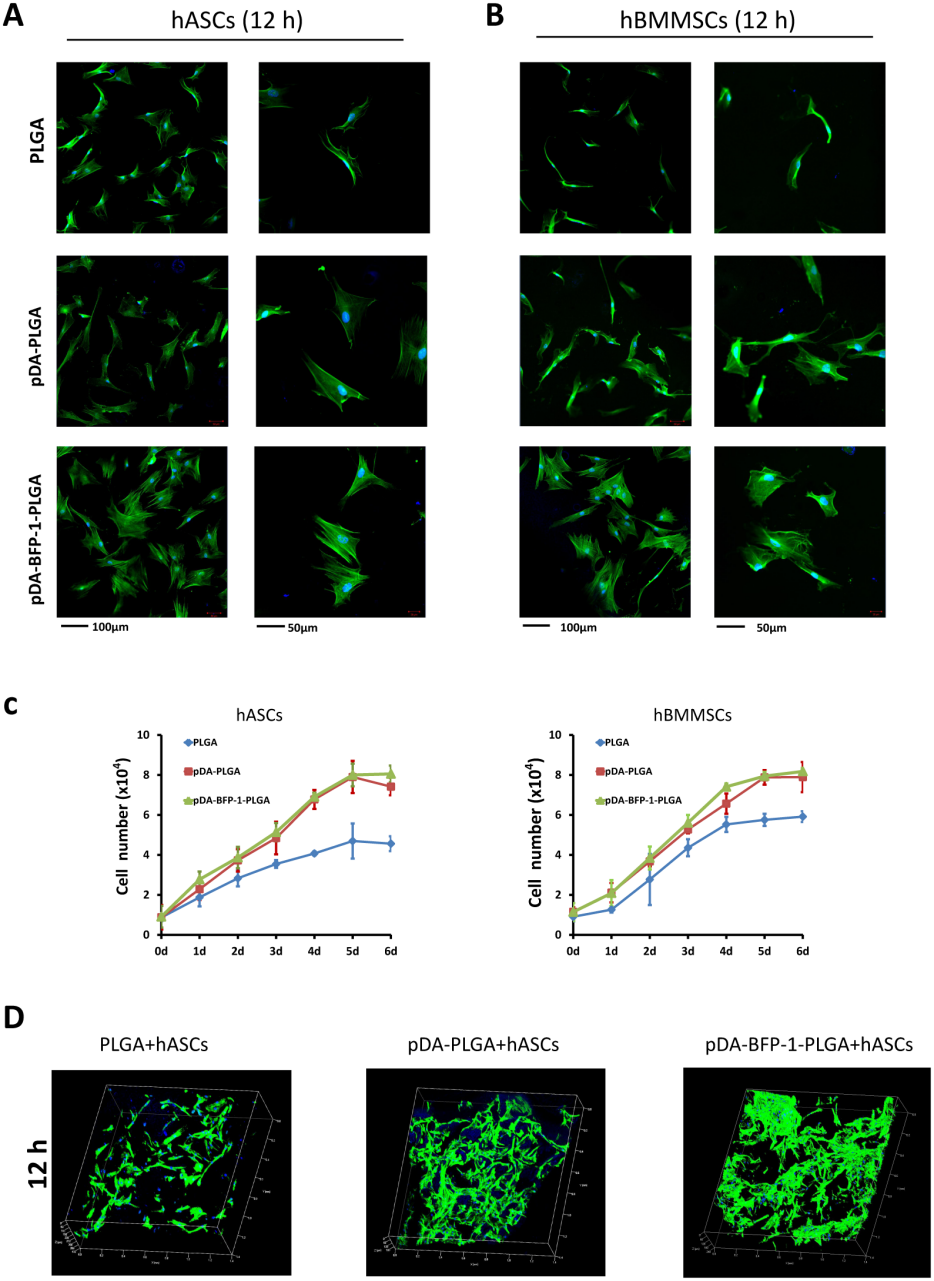
Adhesion and proliferation of hASCs and hBMMSCs on PLGA films. hASCs: human adipose-derived stem cells; hBMMSCs: human bone marrow-derived mesenchymal stem cells. Confocal micrographs of hASCs (A) and hBMMSCs (B) on PLGA, pDA-PLGA and pDA-BFP-1-PLGA films after culturing for 12 h. Phalloidin is colored green and nuclei are colored blue. (C) Proliferation curves of hASCs and hBMMSCs on PLGA, pDA-PLGA and pDA-BFP-1-PLGA film surfaces. (D) Confocal micrographs (50x) of hASCs on PLGA, pDA-PLGA and pDA-BFP-1-PLGA scaffolds after culturing for 12 h. Phalloidin is colored green and nuclei are colored blue. The green signal shown in these pictures indicate the volume of cells.

Fluorescently labeled peptide (FITC-BFP-1) was used to confirm pDA-mediated coating of BFP-1 peptides before investigation of the surface characteristics of the modified PLGA substrates. Peptides could be efficiently grafted to the PLGA surface (Fig 3A), and measurements revealed that the contact angle decreased following modification (60.9°-43.44°-28.1°), indicating a higher hydrophilicity for BFP-1-immobilized PLGA substrates (Fig 3B). X-ray photoelectron spectroscopy (XPS) analysis was used to determine the chemical composition of the modified PLGA surfaces (Fig 3C). Before the modification process, only C1 and O1 peaks were detected on bare PLGA film, whereas after pDA coating, an N1 peak was detected at 399.2 eV, indicating that nitrogen was present at 4.16% (Fig 3D). Following immobilization of BFP-1, the intensity of the N1 peak further increased and to an equivalent of 6.51%, indicating an efficient surface modification. SEM confirmed changes in surface morphology and roughness of the PLGA scaffolds following pDA coating and peptide immobilization (Fig 3E). These structural alterations appeared to facilitate cell adhesion [25], as demonstrated by confocal micrographs of the surface after culturing of hASCs and hBMMSCs for 12 h (Fig 4).

Next, we indirectly calculated the amounts of BFP-1 immobilized on the pDA-PLGA surface by measuring the concentration of unattached BFP-1 in the supernatant. The amount of immobilized BFP-1 was 140.88 ± 7.84 μg on 1.5 cm^2^ of pDA-PLGA scaffolds of 2 mm thickness, and 16.79 ± 5.93 μg on 1.5 cm^2^ of the pDA-PLGA films (Fig 3F). The release of immobilized BFP-1 from PLGA films and scaffolds was measured by calculating the peptide concentration in the supernatant on each day of the 6-day incubation of PLGA substrates in PBS at 37°C (Fig 3G). Fluorescamine assays revealed only 40–50% of the attached BFP-1 had detached from the pDA-BFP-1-PLGA substrates during the 6-day incubation, demonstrating that peptides were stably immobilized. Contact between cells and the surface occurred at the beginning of the process, suggesting the signal generated from the surface was crucial for bone regeneration. To improve cell–biomaterial interactions, two methods have been adopted: modification of the surface of synthetic scaffolds with osteoinductive molecules [26, 27], or enhancing release from the materials [28]. In this study, we immobilized BFP-1 using a bionic method based on poly-dopamine (pDA). Compared with other physical or chemical methods for modification such as physical adsorption or covalent grafting, the pDA-mediated method is easier to control and less damaging to osteoinductive factors. Being only 15 amino acids in length, the peptide avoids the folding or self-assembly that may happen in larger macromolecules. This may increase the binding efficiency of the peptide for the surface by decreasing steric hindrance during the modification process, compared with larger molecules [29].

### Adhesion and proliferation of cells on PLGA substrates

After 12 h of cell culture, confocal microscopy of cells stained with FITC-phalloidin revealed morphological characteristics and facilitated estimation of the number of cells adhered to the surface (Fig 4A and 4B). The number of hASCs and hBMMSCs on pDA-BFP-1-PLGA films was higher than on PLGA films, and cells on this substrate exhibited a more extended polygonal morphology, and this was also the case in scaffold experiments (Fig 4D).

Logarithmic proliferation curves were observed for both hASCs and hBMMSCs on PLGA substrates (Fig 4C). Curves of pDA-PLGA and pDA-BFP-1-PLGA substrates were higher than those observed with PLGA (p <0.05), but differences between pDA-PLGA and pDA-BFP-1-PLGA were not significant. On day 4 of culturing, proliferation on the PLGA film surface reached a plateau, but cells continued to grow slowly on the surfaces of pDA-PLGA and pDA-BFP-1-PLGA. In contrast, proliferation of hASCs and hBMMSCs on pDA-PLGA and pDA-BFP-1-PLGA reached a plateau on day 5. These results are consistent with previous studies in which pDA-coated biological materials promoted proliferation and cell viability [30, 31]. We hypothesize that this may be due to modification of surface topography which initiates transduction of signalling cascades that lead to focal adhesion [32, 33].

### Effect of immobilizing BFP-1 on osteogenic differentiation of hASCs *in vitro*

**Fig 5:**
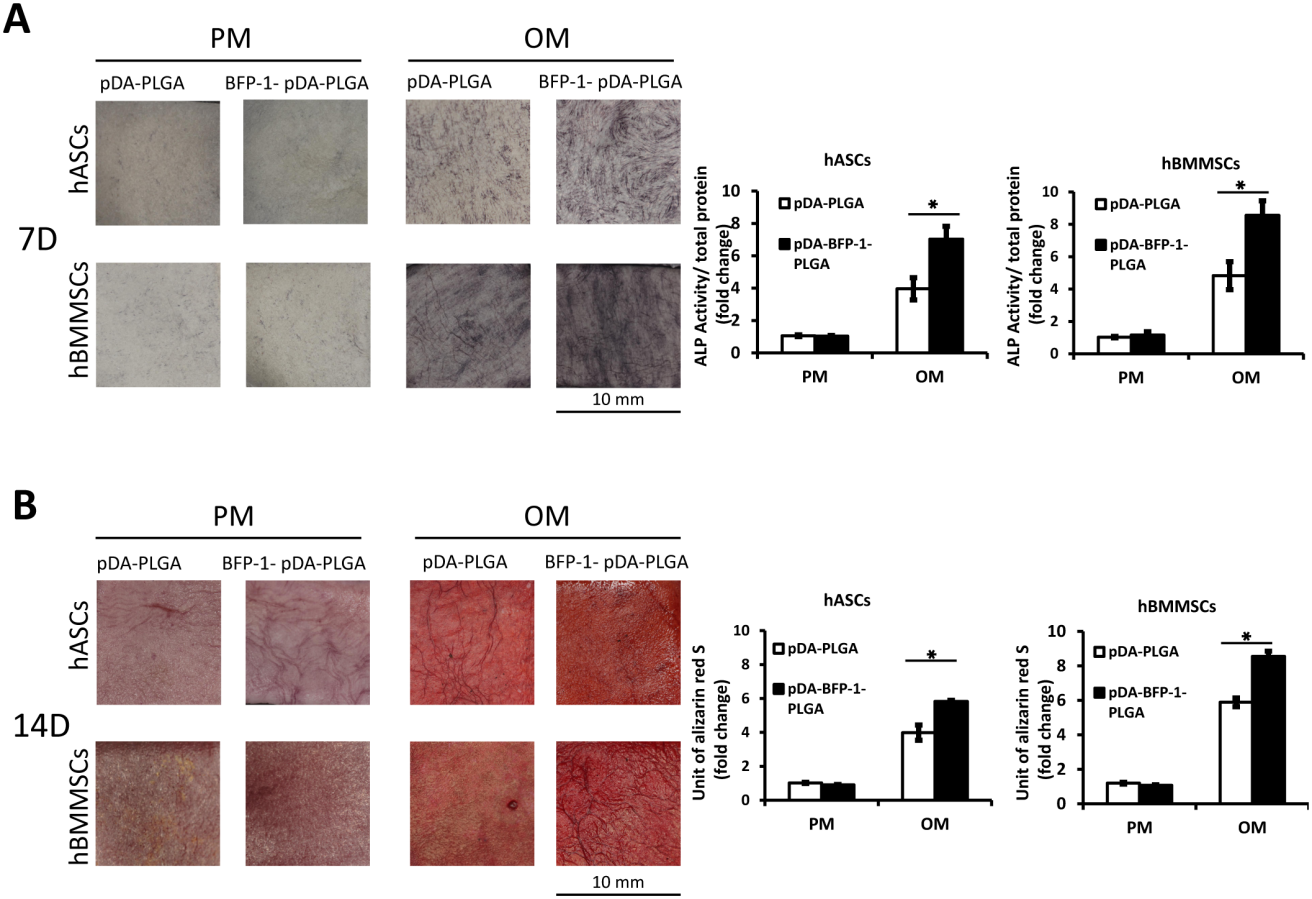
Differentiation of hASCs and hBMMSCs on PLGA substrates *in vitro*. hASCs: human adipose-derived stem cells; hBMMSCs: human bone marrow-derived mesenchymal stem cells. (A) Alkaline phosphatase (ALP) activity of hASCs and hBMMSCs cultured on PLGA films at 7 days after osteoinduction (*: P <0.05). (B) Alizarin Red staining and mineralization assays at 14 days after osteoinduction (*: P <0.05).

To test the osteoinductive activity of engineered PLGA substrates for hASCs and hBMMSCs, ALP activity and mineralization assays were performed. ALP staining and activity were tested after 7 days of osteoinduction (OI), and pDA-BFP-1-PLGA displayed higher activity than pDA-PLGA (P <0.05). In contrast, there was no significant difference in ALP staining or activity in cells that did not undergo OI (Fig 5A). After 14 days of OI, AR-S staining and mineralization assays showed that mineralization was noticeably higher in cells grown on pDA-BFP-1-PLGA (after OI) than controls lacking BFP-1 (p <0.05). Again, there was no significant difference in BFP-1 and control groups in cells that did not undergo OI (Fig 5B).

BFP-1-modified PLGA films showed better osteoinductive ability than non-modified PLGA films, indicating an efficient modification process and suggesting the osteoinductive ability of BFP-1 was not damaged during immobilization. According to the release curve of the immobilized BFP-1 in our study and other previous reports, the connection between the peptide and the poly-dopamine-coated material was stable [34, 35], thus immobilization of BFP-1 to the PLGA surface by the catechol functional group of the poly-dopamine layer is likely stable for a prolonged period and suitable for cell stimulation.

### Histological assessment of bone formation in vivo

For clinical use, it is essential to translate promising *in vitro* results *into in vivo* experiments. We therefore developed three-dimensional PLGA scaffolds loaded with hASCs and implanted them subcutaneously into the back of nude mice. Three materials (PLGA, pDA-PLGA and pDA-BFP-1-PLGA) were tested both with and without cells (six implants in all). After 4 weeks, grafts were white in the PLGA groups but dark in the pDA-PLGA and pDA-BFP-1-PLGA groups, and grafts without cells were of a looser structure than cell-free implants. After 8 weeks, the appearance was as observed as the grafts harvested after 4 weeks, except the cell-free PLGA group in which grafts had disappeared, presumably due to biological degradation [14].

**Fig 6:**
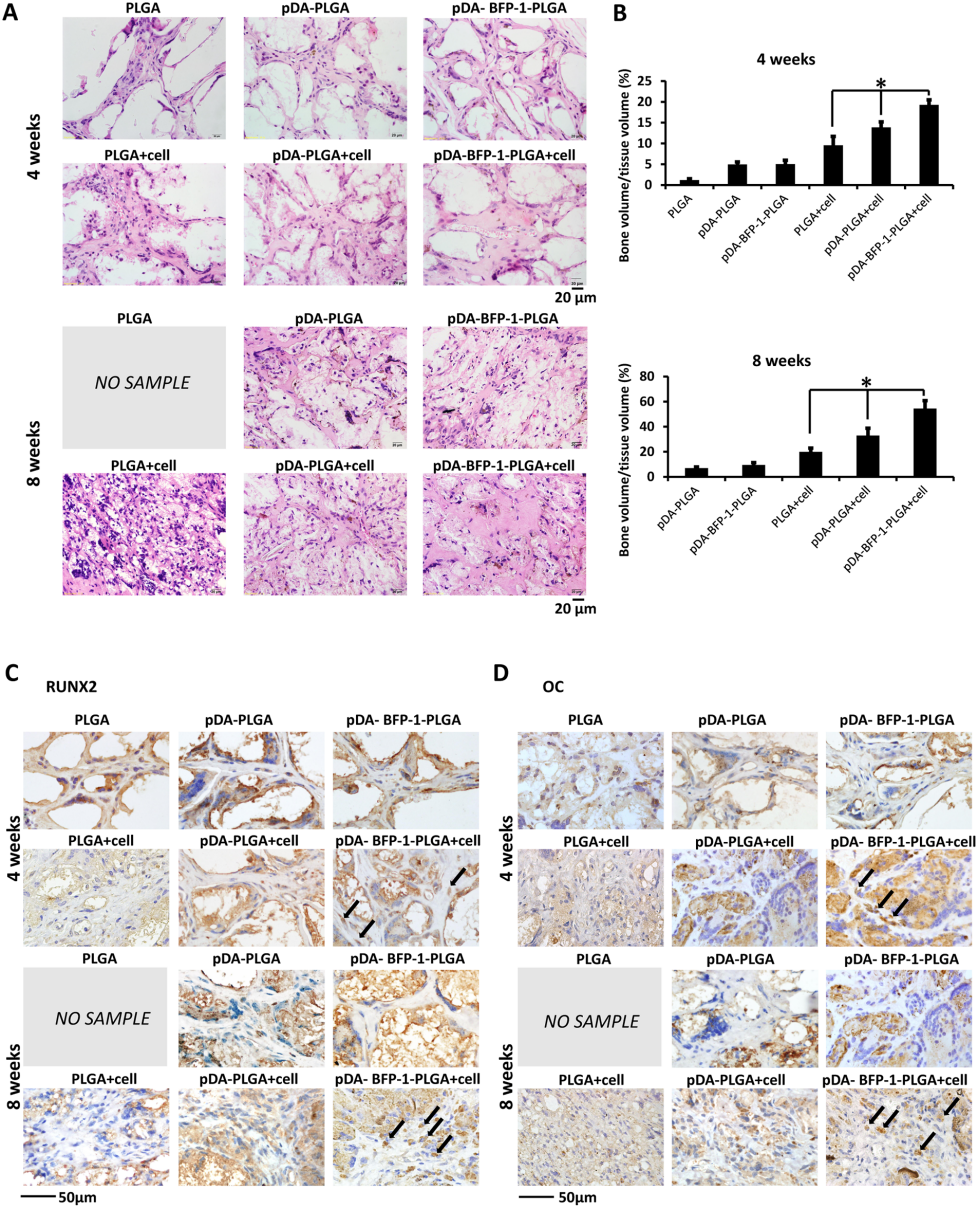
Ectopic bone formation. hASCs: human adipose-derived stem cells. (A) Hematoxylin and eosin (HE) staining. Tissue slices of engineered PLGA scaffolds with or without hASCs were harvested at 4 and 8 weeks after implantation. (B) Semi-quantitative analysis of the staining results. Immunohistochemical staining for RUNX2 (C) and osteocalcin (D).

HE and IHC staining of RUNX2 and OC were used to characterize the newly formed tissue. HE staining of the cell-free PLGA group after 4 weeks revealed that tissue had formed into small cord-like structures, but some fiber-like tissue remained. In contrast, cell-free pDA-PLGA and pDA-BFP-1-PLGA implants displayed a more fibrovascular structure. Implants coated with hASCs resembled eosinophilic bone-like tissues, and had a lower number of cells embedded in the extracellular matrix (Fig 6A). The pDA-BFP-1-PLGA-A group resulted in formation of the largest amount of uniform, acidophilic osteoid tissue, as revealed from staining experiments (Fig 6A). Following IHC staining, a higher proportion of dark brown granules were observed around the nuclei and in the cytoplasm of the group implanted with hASC-impregnated pDA-BFP-1-PLGA compared with other groups, indicating that the osteogenic markers RUNX2 and OC were up-regulated (Fig 6C and 6D). The hASC-impregnated PLGA group had the lowest numbers of these particles, while particles in the hASC-impregnated pDA-PLGA group were intermediate in abundance. Almost no particles were visible in cell-free groups after 4 weeks. After 8 weeks, implants in the cell-free PLGA group had disappeared entirely, but the other groups remained as they were after 4 weeks. Semi-quantitative analysis of the histological results revealed that the hASCs-impregnated pDA-BFP-1-PLGA group clearly exhibited superior ectopic bone formation (Fig 6B *: P <0.05). These results suggest that BFP-1 enhanced osteogenic differentiation of hASCs *in vivo*, and scaffolds modified with BFP-1 possess superior osteoinductive properties.

Three-dimensional scaffolds should be degraded at an appropriate rate if they are to be considered suitable for tissue engineering applications. The biodegradation rate of PLGA materials is dependent on the molecular weight and the copolymer ratio, and the *in vivo* half-live (50:50 ratio of PGA to PLA) was previously found to be 2–4 weeks [14]. Implants are usually degraded faster *in vivo* due to an autocatalytic effect of the accumulated acidic degradation products [14]. In our study, cell-free PLGA implants were completely absorbed 8 weeks after implantation, while implants with surface modifications were still present, suggesting pDA-mediated modification may slow degradation to a rate that is suitable for bone formation. Previous studies reported that cell viability and migration may be negatively affected by acidification of the local environment following rapid degradation of PLGA *in vitro* and *in vivo* [36]. The pDA-mediated immobilization of BFP-1 on PLGA scaffolds may facilitate cellular ingrowth, enhance the osteoinductive properties, and protect against rapid biodegradation.

## Conclusions

BFP-1 displayed similar osteoinductive activity towards hASCs and hBMMSCs, suggesting hASCs can be used in place of hBMMSCs in future bone regeneration research involving BFP-1. Modification of PLGA scaffolds through pDA-mediated immobilization of BFP-1 provides novel materials that possess great promise for bone tissue engineering applications.
